# Assessment of Anti-Cytogenotoxic Effects of Quercetin in Animals Treated with Topotecan

**DOI:** 10.1155/2011/824597

**Published:** 2011-08-15

**Authors:** Saleh A. Bakheet

**Affiliations:** Department of Pharmacology and Toxicology, College of Pharmacy, King Saud University, P.O. 2457, Riyadh 11451, Saudi Arabia

## Abstract

The present investigation was directed to study the possible chemoprotective activity of orally administered quercetin against topotecan-induced cyto- and genotoxicity towards mouse somatic cells *in vivo*. DNA strand breaks, micronuclei formation, and mitotic activity were undertaken in the current study as markers of cyto- and genotoxicity. Oxidative stress markers such as intracellular reactive oxygen species generation, lipid peroxidation, and reduced and oxidized glutathione were assessed in bone marrow as a possible mechanism underlying this amelioration. Quercetin was neither cytotoxic nor genotoxic in mice at doses tested. Pretreatment of mice with quercetin significantly reduced topotecan-induced genotoxicity and cytotoxicity in bone marrow cells, and these effects were dose dependent. Moreover, prior administration of quercetin ahead of topotecan challenge ameliorated oxidative stress markers. In conclusion, quercetin has a protective role in the abatement of topotecan-induced cyto- and genotoxicity in the bone marrow cells of mice that resides, at least in part, on its antioxidant effects. Based on the data presented, strategies can be developed to decrease the topotecan-induced bone marrow suppression and secondary malignancy in cancer patients and medical personnel exposing to topotecan.

## 1. Introduction

Camptothecin, a pentacyclic alkaloid originally isolated from the Chinese plant Camptotheca acuminata by Wall and Wani in 1996 [1], is one of the most important lead compounds in anticancer research. The antitumor activity of camptothecin is thought to be due to its ability to stabilize the reversible covalent DNA topoisomerase I complex [2, 3], preventing the relegation step of the breakage/rejoining reaction mediated by the enzyme. The net result is that the drug causes fragmentation of chromosomal DNA, cell death, extensive sister chromatid exchange, and chromosomal aberrations [4, 5]. Elucidation of the specific target and mechanisms of camptothecin have stimulated intensive efforts to identify novel analogues that overcome the drawbacks of the natural camptothecin molecule, which include low solubility in water; severe and unpredictable toxicity, including hemorrhagic cystitis; reversibility of the drug-target interaction; lactone instability; drug resistance. One of the initial major strategies in this regard has been to improve the solubility of the natural camptothecin by chemical modification [6]. This approach has produced different series of water-soluble analogues or water-soluble prodrugs, among which topotecan (TPT) and irinotecan are the most successful.

The two camptothecin analogues, TPT and irinotecan, have received FDA approval for the clinical treatment of the ovarian cancer and small cell lung cancer [7, 8] and refractory colorectal cancers [9, 10], respectively. Studies focused on these two drugs have demonstrated mechanistic differences between them related to the cytotoxic potency and the stability of the DNA-topoisomerase I cleavable complexes [11]. Consequently, this differential action could influence not only formation of strand breaks during replication but also the likelihood of cell killing and genetic toxicity of these new anticancer agents. Indeed, full therapeutic efficacy of these drugs is limited due to the development of acquired drug resistance by the cancer cells and various side effects in the treated patients, including damages of the normal cells which may lead to the development of secondary tumors. After application of topoisomerase I inhibitors, damage to DNA may result as DNA fragmentation, chromosomal breaks, and micronuclei (MN) formation causing genotoxicity and may lead to carcinogenesis. In animals, TPT is a somatic cell mutagen capable of inducing chromosome aberrations [12]. Follow-up studies of patients who received camptothecins containing regimens revealed an increased incidence of hyperdiploidy and deletion of chromosome 1 [13]. These effects can develop resistance to these therapeutic agents or may lead to the development of secondary tumours. These have prompted the removal of these highly effective agents from some treatment regimens.

In clinical therapy, to increase drug efficacy and decrease the adverse reactions by antitumor agents, it is necessary to combine other drugs [14]. Experimental observations have shown that some flavonoids are able to enhance the cytotoxic action of the chemotherapeutic drugs without damaging normal cells [15]. Consistent with these notions, quercetin, a polyphenolic compound widely distributed in food of plant origin, has been reported to have antitumor effects against several cancer cells [16, 17]. The antitumor effects of quercetin have been reported to induce cell growth inhibition and apoptosis in variety of tumor cells [18]. Quercetin has also been shown *in vitro* to increase the concentration of DNA topoisomerase II inhibiters, doxorubicin, and daunorubicin in some multidrug-resistant cancer cell lines [19]. Moreover, previous reports demonstrated that quercetin increases the growth inhibitory effect of TPT in the treatment of human breast cancer cells [20]. Enhancements of oral bioavailability and reducing gastrointestinal toxicity of irinotecan by quercetin have been also reported [21]. Therefore, the combination of camptothecins with quercetin might be of therapeutic benefit. However, the influence of quercetin on TPT-induced cyto- and genotoxicity in nontumor cells *in vivo* has not been reported yet.

Considering the widespread use of TPT in clinical oncology and the ability of quercetin to improve the therapeutic outcome from TPT prompted to investigate whether quercetin in combination with TPT can ameliorate TPT-induced cyto- and genotoxicity in mice normal tissues. The bone marrow DNA strand breaks, the scoring of MN, and mitotic activity were undertaken in the current study as markers of cyto- and genotoxicity. Oxidative stress markers such as bone marrow reactive oxygen species (ROS), lipid peroxidation, reduced glutathione (GSH), and oxidized glutathione (GSSG) levels were assessed as a possible mechanism underlying this amelioration.

## 2. Results

### 2.1. Effect of Quercetin on TPT-Induced DNA Strand Breaks

The results of alkaline comet assay are shown in Table 1. Quercetin treatment did not exhibit any significant difference in the level of tail moment, tail length, tail DNA, and olive tail moment compared to the solvent control at either dose tested. The positive control cyclophosphamide significantly increases the level of all measured parameters compared to the control group (*P* < 0.01). The results revealed that TPT when given at a single dose of 0.5 or 1 mg/kg causes significant increase in the level of all measured parameters in comparison to those of the solvent control group. However, when pretreatment of different doses of quercetin was given prior to TPT treatment, decreased rates of DNA strand breaks were observed and the higher dose of quercetin gave more effective reduction in all measured parameters.

### 2.2. Effect of Quercetin on TPT-Induced MNPCE and Bone Marrow Suppression

The results of the micronucleus test are presented in Table 2. The frequency of MNPCE in the positive control mutagen cyclophosphamide was significantly higher when compared to the solvent control group (*P* < 0.01). Similarly, TPT at a single dose of 0.5 or 1 mg/kg significantly increased the frequency of MNPCE (*P* < 0.01). Moreover, the mitotic index was significantly decreased after treatment with TPT compared to the solvent control group. Quercetin treatment did not exhibit any significant difference in the frequency of MNPCE compared to the solvent control at both tested doses. In addition, quercetin was not cytotoxic to the bone marrow at the tested doses level. Pretreatment with quercetin was found to significantly decrease the frequency of MNPCE specially at the higher dose of quercetin as compared to the values obtained after treatment with TPT alone. The reduction of mitotic index induced by TPT was found to be restored by pretreatment with the higher dose of quercetin.

### 2.3. Effect of Quercetin on TPT-Induced Oxidative Stress

**Figure 1:**
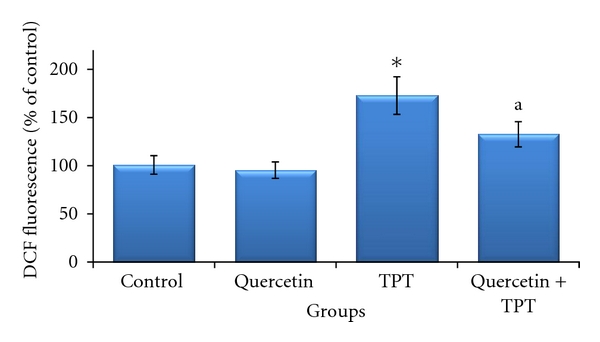
Effects of quercetin (100 mg/kg) on topotecan (TPT; 1 mg/kg)-induced generation of intracellular reactive oxygen species in the bone marrow cells of mice (mean ± SD). *P < 0.05 versus control (Kruskal-Wallis test followed by Dunn's multiple comparisons test), ^a^ *P* < 0.05 versus TPT alone (Mann-Whitney *U* test).

The effect of quercetin on the TPT-induced oxidative stress was assessed by measuring bone marrow ROS accumulation, MDA content, GSH, and GSSG levels. Bone marrow ROS production was evaluated by determining the fluorescent intensity of DCF. As shown in Figure 1, DCF fluorescence level did not show significant variation after treatment of mice with quercetin at 100 mg/kg as compared to the solvent control. The DCF fluorescence level in mice treated with 1 mg/kg TPT was significantly increased by about 1.7-folds as compared to the control animals (*P* < 0.05). However, TPT-induced production of DCF fluorescence was profoundly abrogated by quercetin and decreased to the level significantly different from the level of DCF fluorescence in animals treated with TPT alone (*P* < 0.05).

**Figure 2:**
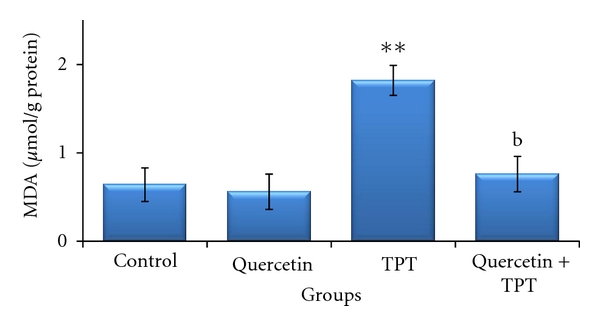
Effects of quercetin (100 mg/kg) and/or topotecan (TPT; 1 mg/kg) on bone marrow lipid peroxidation level (MDA) in mice (mean ± SD). **P < 0.01 versus control; ^b^ *P* < 0.01 versus TPT alone (one way ANOVA and post hoc Tukey-Kramer multiple comparison test).

**Figure 3:**
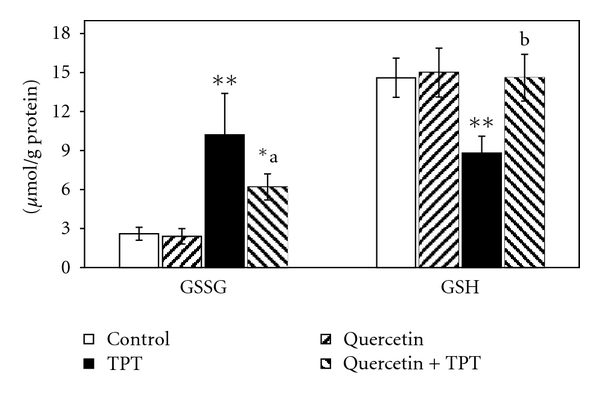
Effects of quercetin (100 mg/kg) and/or topotecan (TPT; 1 mg/kg) on bone marrow oxidized glutathione (GSSG) and reduced glutathione (GSH) levels in mice (mean ± SD). *P < 0.05, **P < 0.01 versus control; ^a^ *P* < 0.05, ^b^ *P* < 0.01 versus TPT alone (one way ANOVA and post hoc Tukey-Kramer multiple comparison test).

**Figure 4:**
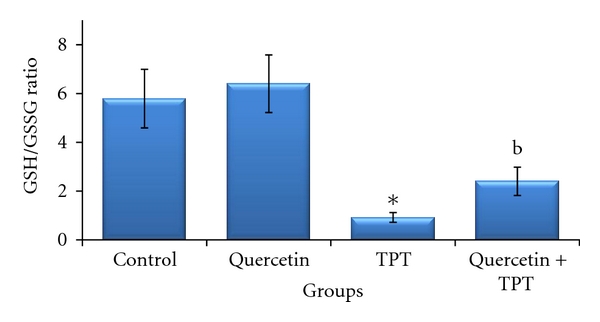
Effects of quercetin (100 mg/kg) and/or topotecan (TPT; 1 mg/kg) on mouse bone marrow reduced and oxidized glutathione (GSH/GSSG) ratio (mean ± SD). *P < 0.05 versus control (Kruskal-Wallis test followed by Dunn's multiple comparisons test), ^b^ *P* < 0.01 versus TPT alone (Mann-Whitney *U* test).

As shown in Figure 2, no significant change in MDA content was observed in bone marrow cells after quercetin treatment in a dose of 100 mg/kg compared to the control. The MDA content in mice treated with 1 mg/kg TPT was significantly increased (*P* < 0.01). The TPT-induced MDA formation was abrogated by quercetin and decreased to the level significantly different from the level of MDA in the TPT treated alone (*P* < 0.01). As shown in Figure 3, bone marrow GSSG and GSH levels did not show any significant variation in 100 mg/kg quercetin-treated animals compared to the solvent control. The GSH level observed in 1 mg/kg TPT-treated animals was significantly decreased, together with increase in GSSG level as compared to the control group (*P* < 0.01); so that GSH/GSSG ratio significantly decreased, indicating increased oxidative stress (*P* < 0.05) (Figure 4). Animals pretreated with quercetin showed a significant increase in GSH level over the 1 mg/kg TPT-treated group and increased to the level significantly different from the level of GSH in the TPT-treated alone (*P* < 0.01). The GSSG level was also significantly decreased in quercetin pretreated animals compared to TPT-treated group (*P* < 0.05). Consequently, the GSH/GSSG ratio was increased in quercetin pretreated animals and was statistically significant when compared to the TPT-treated mice (*P* < 0.01). 

## 3. Discussion

The objective of the current investigation is to determine whether nontoxic doses of the bioflavonoid quercetin, a strong antioxidant present in the human diet, have influence on the cyto- and genotoxicity induced by the anticancer topoisomerase-I inhibitor, TPT, on mice bone marrow cells *in vivo*. The positive control mutagen cyclophosphamide was used in this study, and this compound produced the expected responses. The results of cyclophosphamide were in the same range as those of the earlier studies [22, 23]. These data confirmed the sensitivity of the experimental protocol followed in the detection of DNA damaging effects. The current study demonstrates that quercetin was neither cytotoxic nor genotoxic at the doses tested. Moreover, it is able to protect mouse bone marrow cells against the TPT-induced cyto- and genotoxicity. These results corroborate earlier studies, where oral administration of quercetin did not cause DNA damage in the bone marrow cells [24–26]. 

It was concluded that TPT is a somatic cell cytotoxic and genotoxic. It induced a dose- and time-dependent increases in the percentage of MNPCE and chromosome aberrations in mouse bone marrow after treatment with single doses of TPT [12]. Centromere labelling (FISH assay with the pancentromeric minor DNA probe) showed that about 48% of TPT-induced MN were centromere negative, demonstrating that TPT induces not only chromosome loss but also DNA strand breaks [27]. Both the aneugenic (chromosome loss) and clastogenic (DNA strand breaks) potential of this drug can lead to the development of secondary tumours and abnormal reproductive outcomes.

In agreement with the above-cited report, the present experiment showed that the exposure to TPT caused significant increase in the bone marrow DNA strand breaks and MN frequencies as compared to the values obtained after treatment with the solvent control. Prior administration of quercetin ahead of TPT challenge ameliorated these genotoxic markers and clearly suggests the protective role of quercetin on TPT's genotoxic potentials. The genotoxic protection was also directly correlated with mitotic activity as an obvious protection was noted with quercetin pretreated animals when bone marrow suppression was examined at interphase stage, where, reductions in TPT-induced myelosuppression in mice pretreated with quercetin were observed. Moreover, the protection afforded by quercetin revealed that quercetin could exert dose-dependent anti-cytogenotoxic effects. In fact, the ability of quercetin to confer marked protection against different toxic chemical agents has been described. Quercetin mediated inhibition of bacterial mutagenicity induced by different mutagens [28, 29] and mouse clastogenicity induced by the chemotherapeutic agent; cisplatin has been reported [26].

The exact mechanism by which quercetin protected against TPT-induced cyto- and genotoxicity in bone marrow cells is not well known. One possible explanation for the protection against cyto- and genotoxicity is that simultaneous treatment with quercetin would allow interception of free radicals generated by TPT before they reach DNA and induce cyto- and genotoxicity. In the present work, in order to evaluate whether the observed anti-cytogenotoxic effect was due to an enhancement of the scavenging of free radicals generated by TPT, oxidative stress markers such as ROS accumulation, lipid peroxidation, and GSH/GSSG ratio were evaluated after the animals were treated with TPT, compared with the prior treatment with quercetin and the solvent control animals. The present study demonstrates that quercetin pretreatment reduced the TPT-induced bone marrow ROS accumulation lipid peroxidation and prevented the reduction in GSH/GSSG ratio significantly. 

TPT is able to generate ROS, which causes damage to cellular genome and also the cell membrane leading to lipid peroxidation [30, 31]. The end products of lipid peroxidation also interact with DNA causing DNA strand breaks that in turn develop into chromosomal breaks. These chromosomal breaks may appear as MN in the daughter cell after the first cell division. In agreement with the above-cited reports, the present experiment showed that TPT treatment caused significant increases in ROS and lipid peroxidation levels and quercetin pretreatment reduced the TPT-induced ROS and lipid peroxidation significantly. Quercetin is known as a potent free radical scavenger, capable of inhibiting lipid peroxidation in *in vitro* and *in vivo* systems [25, 26, 32]. 

It has been reported that TPT induces a decrease in the antioxidant enzyme activities in healthy rabbit liver [30, 31]. This can induce cyto- and genotoxicity through the failure of the antioxidant defence mechanisms, since antioxidants are able to protect nontumor cells acting as antigenotoxins without compromising the antineoplastic effects. The increased GSH and GSH/GSSG levels suggest that protection by quercetin may be mediated through the modulation of cellular antioxidant levels. These observations confirm earlier studies in which quercetin was reported to elevate GSH, glutathione peroxidise and superoxide dismutase and to reduce lipid peroxidation [24–26]. A decrease in bone marrow GSH/GSSG ratio noted after TPT-treatment could lead to less protective mechanism in bone marrow cells and thereby developing more TPT induced bone marrow toxicity. However, the group of mice pretreated with quercetin showing decreased cyto- and genotoxic effects and a significant increase in the bone marrow GSH/GSSG ratio suggests the definite significance of GSH also. It could be the elevated level of GSH to protect the cells against TPT-induced cyto- and genotoxicity. 

In summary, a critical point of this study is the possibility that there may be a therapeutic window for the use of TPT in combination with quercetin, so that its harmful side effects in normal cells are minimized. The deleterious effects of TPT might be, at least in part, mediated by an oxidative stress mechanism that may be prevented or reduced by radical scavengers. TPT has a direct effect on DNA topoisomerase I, an important component of its antitumor activity, and this will be unchanged by any manipulations that alter the redox reaction. The improvement in mitotic activity of bone marrow cells of animals pretreated with quercetin in TPT toxicity may focus attention on the beneficial effect of quercetin to overcome one of the most serious problems with cancer chemotherapy, which is the bone marrow suppression and related immunosuppression. Quercetin was effective in reducing cyto- and genotoxicity induced by TPT in bone marrow cells and may possibly decrease the risk of secondary tumors in cells that were not originally neoplastic. The protective effect of quercetin could be possibly ascribed to its radical scavenger effect that modulated the changes induced by TPT. Based on the data presented here, strategies can be developed to decrease the deleterious effects of TPT in normal cells by using quercetin.

## 4. Materials and Methods

### 4.1. Animals

Adult male Swiss albino mice weighing 25–30 g (10–12 weeks old) were obtained from Experimental Animal Care Center, College of Pharmacy, King Saud University. The animals were maintained under standard conditions of humidity, temperature (25 ± 2°C), and light (12-h light/12-h dark). They were fed with a standard mice pellet diet and had free access to water. All experiments on animals were carried out according to the guidelines of the Animal Care and Use Committee at College of Pharmacy, King Saud University.

### 4.2. Drugs and Chemicals

Quercetin (Sigma Chemical Co, St. Louis, USA) was administered by gavage in propylene glycol as a vehicle. Gavages administrations were made 24 h and 1 h prior to the TPT intraperitoneal injection. Control animals were given propylene glycol vehicle only. Quercetin was administered at the doses level of 50 and 100 mg/kg. Upon conversion of animal dose to the equivalent human dose, a dose of 100 mg/kg quercetin in mice was found to be corresponding to 8.1 mg/kg in humans. Accordingly, for an average person weighing 60 kg, 486 mg quercetin would be needed. National dietary record-based cohort assessments of the intake of quercetin from the habitual diet indicated daily levels of quercetin as high as 200–500 mg may be attained by high-end consumers of fruits and vegetables, especially in cases where the individuals consume the peel portion of quercetin-rich fruits and vegetables, such as tomatoes, apples, and onions [33]. TPT and cyclophosphamide (Sigma-Aldrich, St. Louis, USA) were dissolved in saline immediately before use. The doses of TPT (0.5 and 1 mg/kg) were selected on the basis of literature data [12, 27]. All other chemicals were of the finest analytical grade.

### 4.3. Experimental Protocol

Male mice were acclimatized for 2 days and divided into 10 groups consisting of 5 mice each, set up as follows: Group 1: mice were served as a control group and treated daily with the vehicle only for two consecutive days; Groups 2 and 3: mice were treated with quercetin in a dose of 50 or 100 mg/kg, respectively, once a day, for two consecutive days; Group 4: mice were injected with a single dose of 0.5 mg/kg TPT alone; Groups 5 and 6: mice were treated with quercetin at a dose of 50 or 100 mg/kg/day, respectively, once a day, for two consecutive days and 0.5 mg/kg of TPT was administrated on day 2, 1 hour after quercetin exposure; Group 7: mice were injected with a single dose of 1 mg/kg TPT alone; Groups 8 and 9: mice were treated with quercetin at a dose of 50 or 100 mg/kg/day, respectively, once a day, for two consecutive days and 1 mg/kg of TPT was administrated on day 2, 1 hour after regular quercetin exposure; Group 10: mice were injected with a single dose of 40 mg/kg cyclophosphamide and used as a positive control mutagen. 

### 4.4. Detection of DNA Strand Breaks

**Figure 5:**
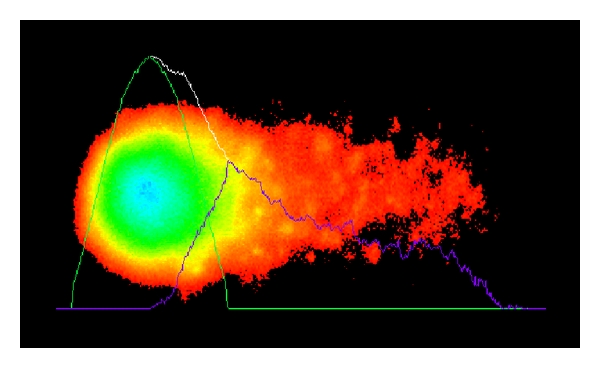
Representative analysis of a fluorescent comet image of a mouse bone marrow cell.

Mice were sacrificed by cervical dislocation 24 h after TPT treatment, and the bone marrow cells from one femur were collected. Single and double DNA strand breaks were studied by alkaline single cell gel electrophoresis (alkaline comet assay) according to the guidelines of Tice et al. [34], with slight modifications as previously described [23]. The slides were stained with ethidium bromide (20 *μ*g/mL) and studied using a fluorescent microscope (Nikon, Japan) equipped with appropriate filters. Fifty individual cells were selected for calculations for each analysis; all experiments were carried out at least three times, each with two parallel slides per data point. Single cells were analyzed with TriTek CometScore version 1.5 software (Figure 5). The parameters studied to access the DNA damage were the tail moment (arbitrary units), tail DNA (%), tail length (*μ*m), and olive moment (arbitrary units).

### 4.5. Bone Marrow Micronucleus Test

The remaining femora from the same animals used for the alkaline comet assay were used for estimation of MN frequencies and mitotic activity. Bone marrow smears were done, and the slides were stained with May-Gruenwald/Giemsa solutions as described earlier [35]. Per animal, 1,000 polychromatic erythrocytes (PCE) were blindly scored microscopically for the presence of MN. In addition, the number of PCEs among 1,000 normochromatic erythrocytes (NCE) per animal was recorded to evaluate bone marrow suppression, mitotic activity was calculated as %PCE = [PCE/(PCE + NCE)] × 100.

### 4.6. Measurement of Oxidative Stress Markers

To study the effect of quercetin on the oxidative DNA damage induced by TPT, animals were treated as in groups 1, 3, 7, and 9. Mice were sacrificed by cervical dislocation 24 h after TPT treatment, and the bone marrow cells from both femurs were collected for estimation of ROS accumulation, lipid peroxidation, GSH, and GSSG levels. The generation of intracellular ROS was evaluated based on the intracellular peroxide-dependent oxidation of 2′,7′-dichlorodihydrofluorescein diacetate (DCFH-DA) to form a fluorescent compound, 2′,7′-dichlorofluorescein (DCF), with modifications as previously described [36]. The bone marrow cells were collected in tubes containing 1.5 mL fetal calf serum then centrifuged and washed with cold PBS (pH 7.4). The bone marrow cells were harvested by centrifugation, washed twice with cold PBS, and finally resuspended in PBS. 200 *μ*L bone marrow cells (2 × 10^5^) were incubated with 200 *μ*L of DCFH-DA (0.4 nM) for 60 min at 37°C in dark. The fluorescence intensity was monitored with a FLUOstar OMEGA microplate reader (BMG LABTECH Ltd., Germany) at an excitation wavelength of 485 nm and an emission wavelength of 520 nm. Results were expressed as fold of control.

Malonodialdehyde (MDA) generated by lipid peroxidation was quantified in the bone marrow cells according to the method of Ohkawa et al. [37], based on thiobarbituric acid (TBA) reactivity. The MDA levels of the samples were calculated from the standard curve using the 1,1,3,3-tetramethoxypropane as the standard and expressed as *μ*mol/g protein. GSH was assayed with 5,5′-dithiobis (2-nitrobenzoic acid) (DTNB) according to the protocol described by Ellman [38]. GSSG was assayed with DTNB, glutathione reductase, and NADPH as described previously [39]. The concentrations of GSH and GSSG were calculated from standard curves that were obtained from freshly prepared standard solutions of GSH and GSSG, respectively, and expressed as *μ*mol/g protein. The value obtained for GSH was divided by the GSSG value to get the GSH/GSSG ratio. Protein measurement was carried out by the method of Lowry et al. [40], using bovine serum albumin as the standard.

### 4.7. Statistical Analysis

Data were expressed as the mean ± standard deviation (SD) of the means. The analyzed parameters were tested for homogeneity of variance and normality and were found to be normally distributed. The data were, therefore, analyzed by employing nonparametric tests, Mann-Whitney *U*-test, and Kruskal-Wallis test followed by Dunn's multiple comparisons test or analysis of variance, ANOVA, followed by Tukey-Kramer for multiple comparisons. Results were considered significantly different if the *P* value was <0.05.

## Figures and Tables

**Table 1:** DNA strand breaks in bone marrow of mice after treatment with quercetin and/or topotecan (TPT) or cyclophosphamide (mean ± SD).

Treatment groups (mg/kg)	Tail moment (arbitrary unit)	Tail DNA (%)	Tail length (*μ*m )	Olive tail moment (arbitrary units)
Control	2.32 ± 0.40	6.78 ± 1.12	16.0 ± 4.84	5.18 ± 1.52
Quercetin (50)	2.68 ± 0.82	6.56 ± 1.67	14.4 ± 5.22	4.34 ± 0.89
Quercetin (100)	2.12 ± 0.35	6.62 ± 2.18	15.2 ± 5.40	3.40 ± 1.11
TPT (0.5)	7.12 ± 1.83**	18.2 ± 3.07**	35.0 ± 9.38*	12.4 ± 3.28*
Quercetin (50) + TPT (0.5)	3.98 ± 1.29^a^	10.2 ± 1.95^b^	24.4 ± 5.22	7.02 ± 1.53^a^
Quercetin (100) + TPT (0.5)	2.64 ± 0.82^b^	7.9 0 ± 2.57^b^	16.8 ± 4.32^b^	5.12 ± 1.66^b^
TPT (1)	13.2 ± 2.27**	22.5 ± 4.42**	50.0 ± 6.44**	18.0 ± 2.51*
Quercetin (50) + TPT (1)	9.54 ± 1.82	14.6 ± 3.54^a^	37.6 ± 4.27^a^	10.9 ± 3.4^a^
Quercetin (100) + TPT (1)	4.28 ± 1.52^b^	11.3 ± 3.90^b^	22.8 ± 6.72^b^	6.88 ± 1.25^b^
Cyclophosphamide 40	23.9 ± 4.57^#^	26.0 ± 6.17^#^	64.2 ± 6.76^#^	14.58 ± 1.93^#^

*P < 0.05 and **P < 0.01 versus control (Kruskal-Wallis test followed by Dunn's multiple comparisons test). ^a^ *P* < 0.05 and ^b^ *P* < 0.01 versus the corresponding TPT alone; ^#^ *P* < 0.01 versus control (Mann-Whitney *U *test).

**Table 2:** Frequency of MNPCE and mitotic activity (% PCE) in bone marrow of mice treated with quercetin and/or topotecan (TPT) or cyclophosphamide (mean ± SD).

Treatment groups (mg/kg)	% MNPCE (mean ± SD)	% PCE (mean ± SD)
Control	0.36 ± 0.11	48.8 ± 1.78
Quercetin (50)	0.32 ± 0.08	48.2 ± 2.15
Quercetin (100)	0.30 ± 0.10	48.4 ± 1.81
TPT (0.5)	1.68 ± 0.24**	41.6 ± 3.43*
Quercetin (50) + TPT (0.5)	0.96 ± 0.23^b^	45.8 ± 2.77
Quercetin (100) + TPT (0.5)	0.58 ± 0.13^b^	47.4 ± 2.40^b^
TPT (1)	2.62 ± 0.54**	39.6 ± 2.19*
Quercetin (50) + TPT (1)	1.90 ± 0.48	44.0 ± 5.78
Quercetin (100) + TPT (1)	0.72 ± 0.14^b^	47.6 ± 2.60^b^
Cyclophosphamide 40	2.06 ± 0.43^#^	42.2 ± 2.58^#^

*P < 0.05 and **P < 0.01 versus control (Kruskal-Wallis test followed by Dunn's multiple comparisons test). ^b^ *P* < 0.01 versus the corresponding TPT alone; ^#^ *P* < 0.01 versus control (Mann-Whitney *U *test).

## Abbreviations

TPT: Topotecan
LMA: Low melting agarose
PCE: Polychromatic erythrocytes
MN: Micronuclei
TBARS: Thiobarbituric acid reacting substance
ROS: Reactive oxygen species
GSH: Reduced glutathione
GSSG: Oxidized glutathione.
